# The Measurements and an Elaborated Understanding of Chinese eHealth Literacy (C-eHEALS) in Chronic Patients in China

**DOI:** 10.3390/ijerph15071553

**Published:** 2018-07-23

**Authors:** Angela Chang, Peter J. Schulz

**Affiliations:** 1 Department of Communication, University of Macau, E21, Avenida da Universidade, Taipa, Macau, China; 2 Institute of Communication and Health, Lugano University, Switzerland, Ex Laboratorio, Via Buffi 13, 6904 Lugano, Switzerland; peter.schulz@usi.ch

**Keywords:** literacy knowledge, health promotion, health status, Internet, mobile use

## Abstract

The rapid rise of Internet-based technologies to disseminate health information and services has been shown to enhance online health information acquisition. A Chinese version of the electronic health literacy scale (C-eHEALS) was developed to measure patients’ combined knowledge and perceived skills at finding and applying electronic health information to health problems. A valid sample of 352 interviewees responded to the online questionnaire, and their responses were analyzed. The C-eHEALS, by showing high internal consistency and predictive validity, is an effective screening tool for detecting levels of health literacy in clinical settings. Individuals’ sociodemographic status, perceived health status, and level of health literacy were identified for describing technology users’ characteristics. A strong association between eHealth literacy level, media information use, and computer literacy was found. The emphasis of face-to-face inquiry for obtaining health information was important in the low eHealth literacy group while Internet-based technologies crucially affected decision-making skills in the high eHealth literacy group. This information is timely because it implies that health care providers can use the C-eHEALS to screen eHealth literacy skills and empower patients with chronic diseases with online resources.

## 1. Introduction

Health literacy has received much attention as a causal agent of health outcomes. For example, low health literacy has been associated with obesity [1,2], low adoption of health behaviours [3,4,5], poor use of health care services [6,7] and poor access to electronic health (eHealth) resources [5]. The rapid rise of Internet-based technologies that disseminate health information and services enhances the acquisition of online health information [8,9,10]. Therefore, eHealth literacy has drawn the attention of researchers as a condition for utilising such information and services [8,11,12].

The present study represents the 1.37 billion people in mainland China who are (as is the rest of the world) undergoing a drastic media communication revolution. Among them, 688 million were Internet users (users) by the end of 2015; of these, a majority of more than two-thirds uses mobile phones [13]. China represents an important case that can reflect how systemic and institutional factors influence and facilitate health communication in the context of rapid media and technological development. Additionally, considering the country’s recent rapid and extraordinary economic growth, it is inevitable that China will eventually encounter the public health problems that developed countries currently experience. Therefore, examination of the relationship between the health status of users and their eHealth literacy is crucial for developing efficient health communication strategies in the future.

### eHealth Literacy

Oh and others [14] analysed 51 unique definitions of eHealth with varying degrees of emphasis on health and computer technology use. The term ‘eHealth’ has been widely used to encompass a set of disparate concepts such as online health management [15] and information searching [16].

Norman and Skinner [17] developed the eHealth literacy scale (eHEALS) based on young users’ (aged < 25 years) eHealth literacy, which was defined as the ability to read, use computers, search for information, understand health information, and put it into context. They understand eHealth literacy as being dependent on basic reading and writing skills. eHEALS is currently the only instrument that aims to measure an individual’s confidence in their ability to locate and evaluate online health information [17,18]. There are six eHealth core skills or areas of literacy comprised in the original Lily model of eHEALS, namely traditional literacy, health literacy, information literacy, scientific literacy, media literacy and computer literacy, the latter measuring working knowledge of computers [17,18,19], in addition to basic understanding of science and appreciation of the social context.

The theoretical grounding and measurement of eHealth literacy has been challenged partially due to the development of social media and users’ increasingly versatile approach to information-finding and problem-solving [20]. Researchers have urged closer examination of the health-related behaviour interventions of adult patients [21,22,23]. Moreover, Macker, et al. [19] reviewed the methodology of eHealth studies and found that a majority of studies tended to employ domain-specific health literacy measures, and concluded that exploratory data collection techniques would improve the quality of research. Another example is in Petrič, et al. [24], who used a revised and extended eHEALS in Slovenia to analyse how patients in online health communities managed their health. To increase the accuracy of eHEALS predictions, Chan and Kaufman [25] proposed a more rigorous theoretical and methodological eHealth framework for characterizing the complexity of eHealth tasks. It could be used to diagnose and describe literacy barriers encountered by participants in performing the tasks.

eHealth literacy is a foundational skill, and eHealth information is a critical predictor of preventive health measures. Chan and others [26] supported the idea that using a problem-based approach to search for reliable health information is effective for increasing the competency level of Chinese university students in Hong Kong. Mackert and colleagues [19] proposed that respondents with high eHealth literacy tended to be younger and more educated than their less eHealth-literate counterparts. A number of other studies confirmed their finding [18,27,28,29,30]. The eHEALS has mainly been administered to young people, who are heavy users, and the participants in studies that use the eHEALS in different countries were also quite health-literate [18,19,31,32]. Reviewing the literature, we concluded that there is a need to recruit adult patients, an overlooked and vulnerable group in online health communication and eHealth research [9,10,33].

The established eHEALS model is a theoretically informed concept with an eight-item baseline for assessing a person’s ability to use eHealth resources [14,15,31,34]. Several studies have criticized the eHEALS model as adopting a unidimensional approach and performing insufficiently in psychometric evaluation [24,35,36,37,38]. However, others have concluded that eHEALS was a valid measurement for predicting health outcomes, and, furthermore, that it improved users’ health communication and the benefit they derive for health care services. For example, Chung and Nahm [39] validated the eHEALS test in English, supporting eHealth interventions to engage older adults in health care and to help them manage their own health. Moreover, Paige and others [33] concluded that eHEALS was a valid and reliable measure of self-reported eHealth literacy among patients with chronic disease in the U.S. Their research supported the use of the eHEALS as part of a screening instrument to help identify patients’ eHealth literacy skills and as a diagnostic tool to define patients who are in need of improving their health literacy.

Several eHEALS are available in a range of languages other than English, including Spanish [31], German [34], Japanese [35], Dutch [36], Italian [37], Portuguese [40] and traditional Chinese [32,41]. The eHEALS measures the use of online health resources and content while considering cultural sensitivities in health information and online communication. However, the considerable body of research on the validity and reliability of eHEALS has been largely limited to the developed Western countries [33,38,39,42]; they have not been examined systematically in simplified Chinese for users in China. Considering China’s recent rapid and extraordinary economic growth, it is inevitable that it will encounter the public health problems currently experienced by the developed countries. 

Our study gave us the opportunity to replicate earlier analyses and generalize findings from other societies and cultures. Therefore, the dual objectives of the present study are first to compare the Chinese eHEALS (C-eHEALS) with other eHEALS findings to determine whether C-eHEALS model provided reliability evidence with chronic disease patients in China. In addition, previous studies have concluded that patients’ access to online health information was affected by their low computer skills and low level of health literacy [33,38]. Thus, the second objective is to examine and compare how the large patient segments access eHealth resources to find and apply eHealth information to their health problems in China. 

## 2. Materials and Methods

### 2.1. Measures and Development of the C-eHEALS

The C-eHEALS expands on previous eHEALS studies of the Chinese in mainland China by including: (1) an eight-item questionnaire baseline for acquiring information from the Internet; (2) a 10-item scale measuring media and computer literacy; and (3) a nine-item literacy scale pertaining to the use of information [14,19,20,33]. The structured questionnaires use a five-point Likert scale (strongly disagree = 1; strongly agree = 5); a higher score indicates a higher eHealth literacy level.

Additionally, we included six questions on socioeconomics and four questions on perceived health status [14,32,33]. The socioeconomics questions were on sex (male or female), age (≥18 years), highest level of education (primary and secondary school, junior high, high school, college or bachelor, master’s degree or higher), residency (Guangdong or others), occupation (business owner, student, self-employed, public servant, clinician or others) and monthly income (<RMB1000, 1000–3000, 3001–5000, 5001–7000, ≥7001). The questions on health status included the frequency of visiting doctors, cost of visiting doctors, and the perceived health condition. Additionally, participants were presented with a list of chronic diseases and required to select all conditions they had, including: cardiovascular disease, metabolic disease, cancer, allergy and other chronic diseases. For the eHealth investigation, online questionnaires were circulated for access via exploratory data collection techniques.

We followed a strict protocol for the translation to ensure that this version was linguistically, technologically and psychometrically robust. The eHEALS was first translated into simplified Chinese to measure respondents’ combined knowledge and perceived skills at finding and applying eHealth information to health problems. The simplified C-eHEALS was then back-translated by a native English-speaker. This did not change any words and all words had equivalent translations. However, two top search tools—an instant messaging app (i.e., Tencent’s Quick Question (QQ)) and a search engine forum (i.e., Baidu) [8]—in the nine-item scale on media and information channel use were specified to allow the questionnaire to be more applicable for the acquisition of health information in China.

### 2.2. Data Collection

The first author and the research team performed the study at a public hospital in Shenzhen, Guangdong, mainland China. The hospital is a major and comprehensive Grade 2A hospital with approximately 2000 employees and 1200 hospital beds. Randomized clinical studies are usually based on convenient sampling [33,38]. In this study, trained interviewers approached patients awaiting admittance at an outpatient department at the hospital. The outpatients were consecutively selected according to their convenient accessibility during the night clinic on every Wednesday and Saturday from October 2015 to June 2016. The C-eHEALS was administered as an online survey via mobile phone, tablet or laptop computer provided by the interviewers or interviewees. The study protocol was approved by the Ethics Committee of the University of Macau (MYRG2015-0123-FSS).

Participants were recruited based on the following inclusion criteria: aged ≥ 18 years, Mandarin-speaking, and a member of the ethnic majority (Han). Respondents were required to select the appropriate answer regarding their comprehension of a series of statements pertaining to the six key eHealth skills. All respondents read an information sheet explaining the purpose of the study before deciding to participate; they provided verbal informed consent for inclusion before their participation in the study began. The C-eHEALS study was tested using a sample of 352 respondents. 

### 2.3. Data Analysis

The Statistical Package for the Social Sciences and Analysis of Moment Structures (AMOS) version 24 (IBM Corp., Armonk, NY, USA) were used in this study. Descriptive analyses and factor analysis were used for an analytical model. For dimensionality reduction from a multidimensional dataset, a pre-determined number of three clusters within the properties of the data was implemented by the K-means algorithm. Cluster analysis of the K-means was used to contrast two groups of low- and high-eHealth literacy. Moreover, a Pearson product–moment correlation coefficient was computed to assess and predict the relationship between the C-eHEALS score and media usage. A chi-square test of independence was performed to examine the relationship between the C-eHEALS scores and users’ demographic information and their reported literacy.

## 3. Results

### 3.1. Identifying C-eHEALS Items

A Kaiser–Meyer–Olkin test (KMO) was used to measure the sample adequacy and conclude the worthiness of factor analysis. The KMO showed a value of 0.928, and the Bartlett test of sphericity was significant ($x^{2}$ = 2814.70, degree of freedom [df] = 28, *p* < 0.001). KMO values > 0.9 are superb, while values between 0.7 and 0.8 are good; KMO values between 0.5 and 0.7 are mediocre [43]. The calculated Cronbach’s α coefficient was 0.954, and the Guttman split-half coefficient was 0.92, indicating good internal consistency for the scale [35,42,43]. Table 1 shows the high internal reliability and validity tests via Pearson’s correlation between the known predictors and the C-eHEALS.

Validity was assessed using factor analysis, which yielded a single-factor solution (eigenvalue = 4.85, 75.81% of the variance explained). The eight items of the baseline C-eHEALS showed high item–total correlation ranging from 0.622 to 0.831, which demonstrates a more valid indication of eHealth literacy skills. Table A1 displays the factor analysis of the C-eHEALS with factor loading and item–total correlation results (Appendix A). 

The inter-item correlation matrix assessed the strength of the eight-item C-eHEALS as well as the direction of the relationship between the two variables for reliability. We found high and positive correlation values, indicating that the items measure the same characteristic. Several factor loadings exceeding 0.7 were indicative of a well-defined structure (e.g., items 2 and 3, and items 4 and 5). Variables with correlation values in the range of 0.3 to 0.4 meet the minimum level for interpretation of structure, values > 0.5 are considered practically significant, and values of 0.7 are considered highly correlated [34,43,44]. Overall, eight factor loadings were classified as excellent (between 0.6 and 0.8). Table 2 shows the baseline eight-item inter-item correlation of the C-eHEALS (*p* < 0.001).

### 3.2. The C-eHEALS Model

**Figure 1:**
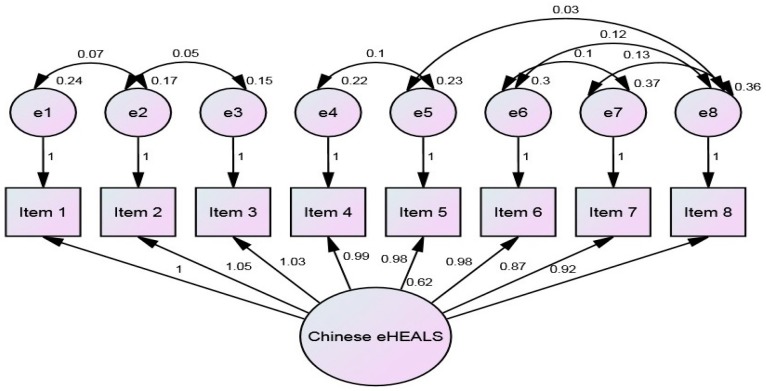
Conceptual model for the relationship between the baseline 8-item of C-eHEALS.

Principal components analysis produced a single-factor solution, and confirmatory factor analysis (CFA) for the baseline eight-item model fit demonstrated high indices (goodness of fit index [GFI] = 0.989, comparative fit index [CFI] = 0.999, root mean square error of approximation [RMSEA] = 0.022, Akaike information criterion [AIC] = 61.292) [43]. Consistent with prior research (e.g., [17,35]) the CFA of the extended C-eHEALS showed a better model fit compared to the original model (GFI = 0.830, CFI = 917, RMSEA = 0.182, AIC = 63.904). Figure 1 depicts the revised conceptual model for the C-eHEALS by AMOS.

### 3.3. Sample Characteristics

The respondents were aged 18–75 years (mean = 28.98 years; SD = 15.53), predominantly male (53.1%; *n* = 187), from the non-Guangdong area (63.2%; *n* = 223) and college-educated (67.6%; *n* = 238). The participants all had chronic conditions. However, the perceived health status was reported as good or very good (41.25%, *n* = 148). In addition, 33 respondents reported that their perceived health status was poor or not good (9.38%). Specifically, 31 respondents reported allergies (8.81%), followed by high blood sugar/pressure (8.24%, *n* = 29), other chronic diseases not listed (6.82%, *n* = 24) and cardiovascular, metabolic diseases or cancer (2.84%, *n* = 10).

From the baseline eight-item C-eHEALS, the group comprising participants aged ≥ 55 years had higher scores (mean = 30.80, SD = 7.04) than the other age groups. A higher mean score was also recorded from male respondents (mean = 29.19, SD = 6.63), post-graduates (mean = 29.43, SD = 5.77) and student respondents (mean = 30.60, SD = 5.50). Table 3 shows the socioeconomic characteristics of the sample and the eight-item C-eHEALS results.

### 3.4. Cluster Analysis of eHealth Literacy

The K-means cluster analysis showed that the high eHealth literacy group had the most frequent agreement regarding the function of sending and receiving emails (61.9%, *n* = 218), while the low–eHealth literacy group had the capability to attach files in email (66.8%, *n* = 235). There were significant differences in the computer skill items between the low- and high-eHealth literacy groups ($x^{2}$ = 615.59, df = 9, *p* < 0.001). Compared to the low-eHealth literacy group, a relatively higher percentage of the high eHealth literacy group worried about computer viruses (61.1%, *n* = 215), were capable of using the Internet to search for information (56.0%, *n* = 197) and could find information online (52.6%, *n* = 185). Table 4 lists a comparison of working knowledge of computer skills and technology literacy categorized in terms of low- and high-eHealth literacy groups (*p* < 0.001).

Compared to the low-eHealth literacy group, the high eHealth literacy group used many eHealth resources to access a greater variety of eHealth content. The reported use of media and information channels related to the C-eHEALS between the low- and high-eHealth literacy groups was significantly different ($x^{2}$ = 703.87, df = 8, *p* < 0.001). To repeat with pre-determined numbers of three clusters within the properties of the data, the K-mean cluster analysis showed that the most frequent use of resources was online encyclopaedias (high-eHealth literacy group: 67.3%, *n* = 237) and face-to-face inquiry (low-eHealth literacy group: 50.9%, *n* = 218). In addition, social media such as QQ were important to the high eHealth literacy group (66.5%, *n* = 234), while the low-eHealth literacy group used mobile phone apps frequently. Table 5 shows a comparison of C-eHEALS media and information channels categorized in terms of low- and high-eHealth literacy groups (*p* < 0.001).

A Pearson product–moment correlation coefficient was computed to assess the relationship between C-eHEALS score and media use. The correlation coefficient for eHealth literacy and computer skill scores was 0.44 (*p* < 0.001). There was a significant association between the C-eHEALS score and personal computer use (r = 0.14, *p* < 0.01) and between the C-eHEALS score and mobile phone use (r = 0.22, *p* < 0.001). In addition, the amount of time spent online correlated significantly with the respondents’ eHealth literacy scores (r = 0.38, *p* < 0.01). Respondents who used the Internet for more than 3.1–5 h per day had the highest eHealth literacy scores.

A chi-square test of independence was performed to examine the relationship between the C-eHEALS scores and online behaviour, taking into consideration information, media and technological literacy. The most significant difference was in the C-eHEALS score and using a mobile phone for online surfing ($x^{2}$ = 370.47, df = 284, *p* < 0.001). A higher frequency of mobile phone use for going online was correlated with a higher level of eHealth literacy. There was a significant relationship between the C-eHEALS score and time spent on social media ($x^{2}$ = 459.90, df = 284, *p* < 0.001). More time spent on social media was correlated with a higher eHealth literacy score. However, there was no significant difference in C-eHEALS scores and use of a personal computer to go online ($x^{2}$ = 289.61, df = 284, *p* = 0.40).

Previous eHEALS studies have examined the association between sociodemographic variables and the use of electronic devices on eHealth literacy levels (e.g., [32,39,42,45]). The present C-eHEALS findings provide further evidence for the idea that eHealth literacy levels are associated with demographic variables such as age and education level. A statistically significant difference was found between eHealth literacy score and age ($x^{2}$ = 326.80, df = 284, *p* < 0.05) and between eHealth literacy score and education ($x^{2}$ = 455.00, df = 284, *p* < 0.001). However, sex, occupation, income, health status and residency were not predictive of the respondents’ eHealth literacy scores. These results could be subject to bias because of the non-representative nature of the respondents.

## 4. Discussion

The C-eHEALS uses the concept of eHealth to assess patients’ comfort and skill in using information technology for health purposes. One of the results supports the idea that the use of computer skills increases health information. To be consistent with previous studies (e.g., [34,35,37,42]), the C-eHEALS is a foundational skill set, a critical predictor of preventive health measures and a validated determinant of various health information–searching behaviours.

The C-eHEALS results also provide an analytical measurement of health literacy by encouraging closer attention to patient age. In line with previous studies (e.g., [17,34,40,45]), the one-factor structure of the C-eHEALS is reliable and internally consistent, with predictive validity among patients in China. The C-eHEALS emerges as an effective screening tool for examining and predicting eHealth use and literacy in clinical settings. 

### 4.1. Low and High eHealth Literacy

Developed in China, the C-eHEALS is comparable to the eHEALS in terms of psychometric properties and predictive ability for patients. In the present study, men, respondents aged > 55 years, respondents with low incomes and respondents who frequently search the Internet had higher C-eHEALS scores. The eHealth user profiles were very different from those in previous studies in developed countries (e.g., [17,37]). However, similarities were also found among the high-health literacy group in the present study as compared to those in previous studies (e.g., [19,33]). The possible explanations are that patients with high C-eHEALS scores tended to use more search strategies to access all types of information online while scrutinizing information more carefully than the respondents with low C-eHEALS scores. The extensive use of the Internet for obtaining health information has highlighted the existence of a high-eHealth literacy group that demonstrates that technologies crucially affect its computer skills. In comparison, we observed that low eHealth literacy levels are potentially associated with poor skills in using a word processor, downloading files, finding health information online, and experiencing difficulty in receiving help from online sources. In summary, high eHealth literacy levels are positively associated with success in using computer skills to find health information and services online.

We found that two items of media and information study (i.e., face-to-face inquiry and websites that provide instant messaging features) were equally important for both the low- and high-eHealth literacy groups. The online resource primarily involved texting, posting, and instant messaging with chat acronyms. To some extent, this could rebut the argument that there is an association between high eHealth literacy and low health literacy in China [45]. The justification relies on the fact that respondents, regardless of eHealth literacy level, considered both face-to-face communication and communication over websites important. In summary, eHealth literacy allows people to acquire the necessary online and offline skills to make informed health decisions.

Those with low eHealth literacy had a high self-perceived interest in face-to-face inquiry, mobile phone app usage, and browsing specific health websites. In comparison, those with high eHealth literacy reported a high use of online encyclopaedias, social media (QQ) and websites with instant messaging communication capabilities. However, the manner in which the low and high eHealth-literate adults perceive the trustworthiness of health information from online health communication channels and information sources is beyond the scope of the present study.

### 4.2. Limitations

Several limitations of this study should be addressed. First, a variety of health-related information and services in China may be found on popular social media sites such as WeChat, Sina Weibo, or Tudou Youku, or on specific health-related platforms (e.g., Keep) [8]. Comprehensive analysis of the current diversity of online media to address hybrid social media issues was not performed. Second, the current eHealth resources are considered merely a means for enhancing human activities or as a substitute in a resource-poor health care environment. Nonetheless, the potentially negative influence and impact of digital media sources and content on users should also be examined [8,18]. Third, we evaluated the properties and the development of the C-eHEALS in a patient population in a selected focus hospital. The cross-sectional design of our study should obtain more sufficient information from patients in a more diverse population from other provincial-level administrative units of mainland China to validate the reliability and validity of the scale. Additional investigations will be required to provide empirical evidence of the generalizability of the scale to other Chinese population samples. 

## 5. Conclusions

C-eHEALS is a valid and reliable measure of self-reported eHealth literacy among chronic disease patients in China. This information is timely because it implies that health care providers can use the C-eHEALS to screen eHealth literacy skills and empower patients with chronic diseases with online resources. Moreover, a strong association between eHealth literacy level, media information use, and computer literacy was found. To be specific, Internet-based technologies crucially affected decision-making skills in the high eHealth literacy group while the emphasis of face-to-face inquiry for obtaining health information was important in the low eHealth literacy group. Although Internet and other digital technologies mediate eHealth communication and health promotion efforts, extensive research is required to determine whether the technical advantages of eHealth communication can be effective within the social reality of the diverse means by which people communicate and perceive reliable health content. An improved follow-up implementation using controlled intervention for evaluating web-based eHealth programs in a specific clinical environment should also be considered. Increased eHealth literacy should be examined as an important determinant of a range of health-related behaviours and an important predictor in the utilization of preventative health measures.

Chinese patients frequently access health information via social media, online encyclopaedias and websites that provide instant messaging features. The present study reflects the influence and features of the aforementioned online resources that facilitate health information acquisition. One of the practical implications is that health care providers must be aware of their patients’ eHealth literacy levels to maximize the benefits of eHealth technologies in the digital era. Furthermore, the knowledge and skillsets required for eHealth literacy must be increased by improving certain levels of computer competency. 

## Figures and Tables

**Table 1:** Results of reliability and validity tests.

Reliability		Validity				
Cronbach’s		Media & Computer	Computer	Information	Health	Education
alpha	Split-half	literacy	skills	literacy	status	attainment
0.95 ***	0.92 ***	0.13 *	0.44 ***	0.12 *	0.08	0.11 **

*** *p* < 0.001, ** *p* < 0.01, * *p* < 0.05.

**Table 2:** Inter-item correlation of the C-eHEALS.

Item	1	2	3	4	5	6	7	8
1. I know what health resources are available on the Internet	1							
2. I know where to find helpful health resources on the Internet	0.839	1						
3. I know how to find helpful resources on the Internet	0.780	0.869	1					
4. I know how to use the Internet to answer my questions about health	0.708	0.764	0.777	1				
5. I know how to use the health information I find on the Internet to help me	0.703	0.760	0.754	0.852	1			
6. I have the skills I need to evaluate the health resources I find on the Internet	0.698	0.721	0.722	0.709	0.73	1		
7. I can tell high quality health resources from low quality health resources on the Internet	0.635	0.660	0.668	0.635	0.656	0.725	1	
8. I feel confident in using information from the Internet to make health decisions	0.638	0.671	0.688	0.669	0.706	0.769	0.73	1

Note: Significant at the *p* < 0.001 probability level for all cells (two-tailed test).

**Table 3:** Socioeconomic analysis and level of eHealth literacy in China.

Variables		*n* (%)	8-Item Score
		352 (100)	Mean ± SD
Sex	Male	187 (53.1)	29.19 ± 6.63
	Female	165 (46.9)	28.75 ± 6.27
Age	18–25	124 (35.2)	29.84 ± 6.79
	26–35	137 (38.9)	28.64 ± 6.26
	36–45	56 (15.9)	28.39 ± 6.16
	46–55	30 (8.5)	27.80 ± 6.27
	Over 55	5 (1.4)	30.80 ± 7.04
Education	Primary & secondary school	2 (0.6)	20.00 ± 5.66
	Junior high	9 (2.6)	28.67 ± 3.64
	High school	22 (6.3)	25.73 ± 7.67
	College or bachelor	238 (67.6)	29.22 ± 6.55
	Master degree or above	81 (23.0)	29.43 ± 5.77
Resident ***	Guangdong	129 (36.6)	28.63 ± 6.60
	Others	223 (63.2)	29.46 ± 6.48
Occupation	Business	180 (51.5)	28.74 ± 6.54
	Student	63 (17.9)	30.60 ± 5.50
	Self-employed	34 (9.7)	29.60 ± 6.45
	Public servant	29 (8.2)	29.96 ± 4.82
	Clinicians	27 (7.7)	26.25 ± 5.33
	Others	19 (5.4)	24.95 ± 9.36
Income (RMB)	less than 1000	55 (15.6)	29.35 ± 5.55
	1000–3000	50 (14.2)	29.32 ± 6.69
	3001–5000	85 (24.1)	28.61 ± 6.94
	5001–7000	75 (21.3)	28.59 ± 6.49
	7001 & above	87 (24.7)	29.26 ± 6.44

Note: *** significant at the *p* < 0.001 probability level.

**Table 4:** Reported computer and technology skills of the C-eHEALS by groups of low and high eHealth literacy.

Item	Low eHealth *n*	%	High eHealth *n*	%
1. Able to attach files in email	235	66.8	71	20.2
2. I worry about computer virus	62	17.6	215	61.1
3. My computer skills are better than my peers	43	12.2	73	20.7
4. Have knowledge about intellectual property	33	9.4	98	27.8
5. Can use a computer to do my work	25	7.1	159	45.2
6. Know how to use a word processor	24	6.8	160	45.5
7. Can send and receive email	20	5.7	218	61.9
8. Can use the Web to search for information	19	5.4	197	56.0
9. Can find the file on my computer after downloading	19	5.4	174	49.4
10. Can find information on the Web	12	3.4	185	52.6

**Table 5:** Reported media and information channels of the C-eHEALS by groups of low and high eHealth literacy.

Item	Low eHealth *n*	%	High eHealth *n*	%
1. Face-to-face inquiry	179	50.9	73	20.7
2. Mobile phone apps	72	48.9	19	5.4
3. Specific health websites	167	47.4	28	8.0
4. Hospital website	160	45.5	27	7.7
5. Online forum	125	35.5	37	10.5
6. Websites with instant feedback	77	21.9	205	58.2
7. Social media (e.g., QQ)	65	18.5	234	66.5
8. Online Encyclopedia	45	12.8	237	67.3
9. Search engine (e.g., Baidu)	19	5.4	155	44.0

## Appendix A

**Table A1:** Factor analysis of the C-eHEALS with factor loading and item–total correlation results.

Item	Factor Loading	Communalities	Item-Total Correlation	α, If Item Deleted
1. I know what health resources are available on the Internet	1	0.744	0.730	0.949
2. I know where to find helpful health resources on the Internet	0.839	0.819	0.831	0.945
3. I know how to find helpful resources on the Internet	0.780	0.812	0.798	0.946
4. I know how to use the Internet to answer my questions about health	0.708	0.773	0.772	0.947
5. I know how to use the health information I find on the Internet to help me	0.703	0.785	0.777	0.947
6. I have the skills I need to evaluate the health resources I find on the Internet	0.698	0.759	0.713	0.948
7. I can tell high quality health resources from low quality health resources on the Internet	0.635	0.665	0.622	0.952
8. I feel confident in using information from the Internet to make health decisions	0.638	0.707	0.681	0.950

Note: eigenvalue = 4.85; cumulative variance explained 75.81%.
